# The effect of productive SafetyNet program on wasting among under-five children in the rural community of South Gondar Zone, Northwest Ethiopia

**DOI:** 10.1186/s13690-020-00481-4

**Published:** 2020-10-12

**Authors:** Melaku Tadege Engidaw, Alemayehu Digssie Gebremariam

**Affiliations:** Public Health Department, College of Health Sciences, Debre Tabor University, P.o.Box: 031, Debre Tabor, Ethiopia

**Keywords:** Productive SafetyNet program, Wasting, Associated factors, Under-five children, Ethiopia

## Abstract

**Background:**

Undernutrition is a significant public health problem in a developing country like Ethiopia. Even if the cause of malnutrition is multifactorial, it is mainly related to socioeconomical, political, and health-related problems. All these problems will lead to more severe nutrient deficiencies among households without assets. The Productive SafetyNet program is implemented for beneficiaries in the rural community to prevent household assets depletion. So, this study aimed to assess the effect of a Productive SafetyNet program on wasting among under-five children in the rural community of South Gondar Zone.

**Methods:**

A community-based cross-sectional study was done among 803 children paired with their mother/caregivers. Particepnts were selected by a simple random sampling technique. The data were collected by using a structured and pre-tested questionnaire. AnthroPlus software was used to analyse anthropometric data. The data entry employed by EpiInfo version 7.0 software for Windows. Then, the data exported to SPSS version 20.0 to carryout further statistical analysis. The anthropometric index (weight for height) was constructed to determine wasting. Both binary and multivariable logistic regression models were used to identify associated factors. Finally, *P*-Value ≤0.05 was used to declare statistical significance.

**Results:**

In this study, the response rate was 95.76%. Of the total participants, 195(25.36%) children were from SafetyNet program beneficiaries. The overall prevalence of wasting was 29.9% (95% CI: 26.6, 33.2%). More than One-fourth of the children from SafetyNet beneficiary households were wasted. While considering all other variables constant, Productive SafetyNet Program reduce wasting by 46% (COR = 0.54. 95% CI (0.37, 0.79)). Wasting were significantly associated with marital status (divorced and/or separated: AOR = 3.33, 95% CI (1.71, 6.45)), being on the SafetyNet program (AOR: 0.63, 95% CI (0.40, 0.99)), family size (AOR = 0.13, 95% CI (0.09, 0.21)), father educational status (AOR: 0.25, 95% CI (0.09, 0.66)), age of the child (AOR = 0.51, 95% CI (0.33, 0.77)), and child dietary diversity score (AOR = 2.99, 95% CI (1.67, 5.35)).

**Conclusion:**

Wasting was a severe public health problem. In this study, the Productive SafetyNet Program reduce wasting significantly. Marital status, SafetyNet program status, family size, father educational status, age of the child, and dietary diversity were factors associated with wasting among children. Early detection of household asset depletion and SafetyNet program implementation is vital with the usual nutritional assessment and counseling.

## Background

The nutritional status of children is an indicator of economic growth, a reflection of the household’s living standard, and child survival according to Millennium Development Goal one [1]. Even if Ethiopia has the highest population growth, currently, parallel economic growth, expansion of the services, and the agricultural sector’s productivity play a vital role to reduce poverty [2]. With this economic growth, poverty, and food insecurity are still the main problem due to ineffective & inefficient agricultural marketing system, underdeveloped transport, communications, and production technologies, limited access to rural households to support services, and environmental degradation which affects their livelihoods [3, 4].

Since 2005, the Productive SafetyNet program (PSNP) was implemented in Ethiopia as a new approach to address chronic food security through transfers to chronically food-insecure households to prevent asset depletion. In 2009, PSNP+ was launched to connect and enhance small income-generating activities by households through financial services and markets to have a self-sustainable finance [5–7]. Millions have enrolled into PSNP to meet consumer needs, reducing the risks they faced and providing them with alternative options to selling productive assets because of food insecurity [5].

Still today, household food insecurity, hunger, and undernutrition remain critical issues and persistent problems in Ethiopia. Also, undernutrition (like Wasting, Stunting, Underweight, and anaemia) is a public health significant problem in each region [8–11]. Amhara regional state is the one with the highest-burden of undernutrition, especially with the highest prevalence of poor nutritional status of women and children [12, 13]. Mainly, inadequate dietary intake, lack of dietary diversity, lack of nutrient density in the food, inappropriate feeding practices, and lack of hygienic practices are the cause of poor nutritional status among children. In addition to these, illness leads to failure to take and absorb adequate essential nutrients for their growth & development [14].

There is a high prevalence of stunting, underweight, and wasting among children with PSNP dependent households as compared to those households without PSNP in Ethiopia [13]. Food insecurity leads to the poor nutritional status of children and decreasing productivity. This may follow the intergenerational life cycle and affects both the economic growth and development of the country at large. Food insecurity affects the school’s attendance and educational attainment of adolescents in Southwest Ethiopia [15].

Even if undernutrition is a public health problem in Ethiopia, in this research, we are interested to assess the effect of the PSNP on wasting. The aim of the PSNP’s in Ethiopia was to increase the percentage of children age 6 to 23 months to have access to minimum acceptable diet groups, household dietary diversity, the average value of asset holdings, and the number of different income sources. All these indicators show that PSNP has a positive effect on the current intake and avoid the risk of food insecurity [16]. The objective of the current study is to assess the effect of the PSNP on wasting among under-five children in the rural community of South Gondar Zone.

## Materials and methods

### Study design and period

A community-based cross-sectional study was employed from December 01 to January 30, 2017.

### Study area

The study was conducted in the selected Woredas of South Gondar Zone. There are five Woredas/districts with a PSNP (LiboKemkem, Simada, Lay Gayint, Tach Gayint, and Ebnat) in South Gondar Zone. Among these, Simada and LiboKemekm Woreda were selected by lottery methods. In these districts, agricultural activities were limited to a single rainy season (from June to September). Maize, barley, and millet are the main food crops. Also, white rice, vetch, and chickpeas are the main cash crops. The number of households in the Woredas with a PSNP was more than 9000 (to public work and direct supports). The total number of children less than 5 years was 34,986. Among these, near to 20,000 (459 were selected) and 15,000 (344 were selected) children were from Libo – Kemkem and Simada Woredas respectively.

### Eligibility criteria

All rural children from 06 to 59 months of age with their caregiver/parents in Libo – Kemkem and Simada Woreda’s during data collection periods were a part of this study. But, guest children and did not stay > 6 months in the area were excluded.

### Sample size determination

A single population proportion formula used to determine the sample size by taking a confidence level of 95%, the marginal error of 5%, and the prevalence of wasting was 38.7% from a study conducted in Wondogent, Sidama Zone, Ethiopia [17]. The final sample size was 803 children paired with their mother or caregivers after adding a design effect of 2 and 10% non-response rate.

### Sampling procedure

A multistage sampling method was employed to select all the study participants from Libo – Kemkem and Simada Woredas. In Libo – Kemkem Woreda there were near to 20,000 children, 459 were included in this study and from Simada 344 children from near to 15, 000. Nine and eight Kebeles were selected from Libo-Kemekem and Simada Woreda respectively by using simple random sampling (random table) after listing all accessible rural kebeles alphabetically (A to Z).

Then, the proportional allocation of the sample size to each kebele was made. The required sample size of 06–59 months was selected by using a simple random sampling technique after obtaining the list of each child in the family folder. During data collection, single children from 06 to 59 months were selected by lottery method from the household in case of two or more children within a single household were obtained.

### Measurements and tools for data collection

Well-structured and pre-tested Amharic version questionnaires were used. The questionnaire had variables related to socio-economic, anthropometrical, maternal obstetric health, and dietary habits of the children. Except for anthropometrical data, all the others were collected from the mother/caregiver, including the dietary history (foods consumed by the child within the past 24 h), socioeconomic, and maternal obstetric health-related variables.

The Anthropometric data were collected by using BSc Nurses. A total of 3 individuals per team was participated to collect the data. While one person records the data, the others took the height and weight. Here, standardization was done for each measurement. The child weight measured by using a calibrated electronic Seca scale (made in Germany). This device is accurate to 0.1 kg. The child height measured three times with a Stadiometer, accurate to 0.1 cm. Mid upper arm circumference (MUAC) was measured on the left arm for right-handed or vice versa by inserting arm circumference tape accurate to 0.1 cm using meter tape distributed by UNICEF for this purpose.

The weights were measured after take-off the shoes and wearing a possible light close. For children less than 2 years, we were using the panty bag to measure the weight. The wooden height or length board used to take the height (> 2 yrs) or the length (< 2 yrs) for children’s interchangeably. The height/length was measured by placing the participants into the Frankfurt position.

### Data quality

The questionnaire was prepared in the Amharic language to make it simple and easily understandable. Nine data collectors (a team of three individuals), three supervisors, and principal investigators were involved in the data collection process after 02 days training on how the data collectors and supervisors interview the mothers, fill the questionnaire, and taking physical measurements by using standard instruments of weighing scale and height/length board. The pre-test was done on 20 children from adjacent Woredas before actual data collection. After the pre-test, necessary correction and modification were done.

To get the appropriate age, we reduced recall bias by using prominent local events, Baptism cards, and immunization cards. Weighing scales were calibrated by using 1 kg of standard weight before each measurement. Three consecutive measurements were taken to ensure accuracy. Finally, the average score of the three measurements used to determine the child’s height and weight. Weight and length/height were recorded into the nearest 0.1 kg and 0.1 cm respectively. All collected questionnaires were reviewed every night by the supervisors and investigators to check completeness and consistency. Then, the feedback was given to the data collectors to handle the problems faced during data collection.

### Definitions of terms

**Wasting:** when a child’s weight for height Z-score was <−2SD of the median value of the NCHS/WHO curve [18].

**Underweight** - when a child’s weight for age Z-score was <−2SD of the median value of the NCHS/WHO curve [18].

**Household**: People who sleep under the same roof and take meals together at least 4 days a week.

**Households with PNSPs**: households identified as chronically food insecure and currently under a cash transfer or asset-building program [19, 20].

**Kebele**: is the smallest administrative division or village in Ethiopia.

**Dietary diversity score (DDS)**: is the consumed food by the child within 24 h and was categorized as low (consumed < 4 food groups) and good (consumed ≥4 food groups) dietary diversity scores [21].

### Data processing and analysis

The age, sex, height/length, and weight of children entered into the WHO AnthroPlus software to build anthropometric indices based on the growth reference of NCHS to compute anthropometric indices. During WFH analysis, all flagged cases were excluded from this analysis. All the data entered by using EPI info version 7.0 and then exported into SPSS version 20.0 for windows for cleaning and analysis. Both bivariable and multivariable logistic regressions were done to identify the associated factors of wasting. Then, COR and AOR with 95% CI were used to see the levels of significance of the association. Finally, p valve ≤0.05 was used to declare statistical significance.

## Results

### Socio-demographic characteristics

The response rate for this study was 95.76%. The average age ± SD the respondent was 30.11 ± 5.16 years and the majority of the respondents (267(34.7%)) found between the ages of 25 to 29 years. All of them were Orthodox by religion. One-fourth of the households are under the SafetyNet program. More than ¾ of the mother and caregiver (591(76.9)) can read and write. The average age ± SD the child was 31.30 ± 20.51 months and near to 55% found between 23 and 59 months of age’ (Table 1).

**Table 1:** Socio-economic characteristics of the respondent to the effect of PSNP on wasting among children 06–59 months of age in selected Woredas of South Gondar Zone Ethiopia, 2017 (*n* = 769)

Variable	Categories	Frequency	Percent
Current maternal age (completed years)	15–19	12	1.6
	20–24	76	9.9
	25–29	267	34.7
	30–34	252	32.8
	35–39	107	13.9
	40–44	55	7.2
	45–49	09	1.2
Household head	Husband/brother^a^	734	95.4
	Wife/Sister^a^	35	4.6
Enrolled in the PSNP	Yes	195	25.4
	No	574	74.6
Marital status	Single	59	7.7
	Married	630	81.9
	Separated	80	10.4
Respondent’s educational status	unable to read and write	591	76.9
	able to read and write	178	23.1
Husband’s educational status	unable to read and write	427	55.5
	able to read and write	314	40.8
	primary education and above	28	3.7
Respondent’s occupation	Housewife	385	50.0
	Daily labourer	35	4.6
	Farmer	349	45.4
Husband’s occupation	Daily labourer	36	4.7
	Farmer	733	95.3

^a^this brother or sister is for orphanage children; these children caregivers will be married or not

### Obstetrics related characteristics

The average age difference between the index and the next child ±SD was 3.20 ± 1.54 years. The majority of the mothers and caregiver (592 (77.0%)) started complementary feeding at the age of ≥06 months. Near to 5% of the mother gives extra food/beverages after delivery instead of colostrum. Of all deliveries, 85% of the children delivered at health institutions (Table 2).

**Table 2:** Obstetrics related characteristics of the respondent to the effect of PSNP on wasting among children 6–59 months of age in the selected Woredas of South Gondar Zone, Ethiopia, 2017 (*n* = 769)

Variable	Categories	Frequency	Percent
The age difference between the last two children	< 2 years	283	36.8
	≥2 years	486	63.2
Breastfeeding status	Yes	744	96.7
	No	25	3.3
Age to start complementary feeding	Immediately after birth	59	7.7
	01–06 months	118	15.3
	≥6 months	592	77.0
Giving additional/other foods or beverages after delivery	Yes	37	4.8
	No	732	95.2
Bottle feeding	Yes	68	8.8
	No	701	91.2
Attending ANC for current child	Yes	697	90.6
	No	72	9.4
Place of delivery	Health institution	654	85.0
	Home	115	15.0
Vaccination history	Yes	720	93.6
	No	49	6.4
Illness in the last 2 weeks	Yes	193	25.1
	No	576	74.9
Hand washing practice	Yes	656	85.3
	No	153	14.7

### The magnitude of wasting and dietary related characteristics

**Fig. 1:**
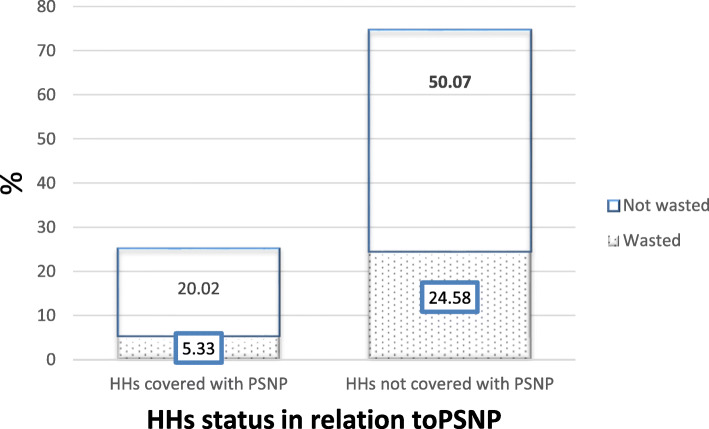
The percentage of wasting from households covered and not covered with PSNP among 06–59 months of age children in the selected Woredas of South Gondar Zone, Ethiopia, 2017 (*n* = 769)

The mean age ± SD was 23.38 ± 13.20 months. The mean ± SD of weight and height of the child was 10.05 ± 2.54 Kg and 81.12 ± 14.49 cm respectively. The mean ± SD of MUAC was 13.24 ± 0.92 cm. In this study, the number of children with a MUAC ≤11.5 cm was 06(0.78%). The mean ± SD of WFH z score was − 0.79 ± 1.98. The prevalence of wasting was 29.9% (95% CI: 26.6, 33.2%) and from this, 16.5% (95% CI:13.8, 19.2%) were very severe. Of the total participants, 195 (25.36%) children were from SafetyNet program beneficiaries (Fig. 1).

The average dietary diversity score ± SD was 5.23 ± 1.14 by using a 24-h dietary recall. Most of the children (678 (88.2%)) had a good dietary diversity score.

### Factors associated with wasting

While all other factors were assumed constant, being a part of a PSNP household family member reduces the prevalence of wasting by 46% (COR: 0.54, 95% CI (0.37, 0.79).

Also, the combined effects of the variables were assessed by bivariable and multivariable binary logistic regression models. In bivariate logistic regression; marital status, the age of the child, the sex of the child, family size, enrolled in the PSNP, father educational status, husband’s occupation, maternal occupation, child’s dietary diversity score (CDDS), history of ANC follow-up, birth interval, and vaccination history were the significant variables.

Finally, marital status, father educational status, being on PSNP, family size, age of the child, and child DDS were the associated factors for the development of wasting among children aged 6–59 months during multivariable binary logistic regression (Table 3).

**Table 3:** Factors associated with wasting among children aged 06–59 months in selected Woredas of South Gondar Zone Ethiopia, 2017 (*n* = 769)

Variable	Wasting		COR (95% CI)	AOR (95% CI)
	Yes	No		
Marital status				
Married	186	444	1	1
Single	7	52	0.32(0.14, 0.72)	0.63(0.25, 1.56)
Divorced and separated	37	43	2.05(1.28, 3.29)	**3.33(1.71, 6.45)** ^**a**^
Age of the child				
6–11 months	44	982	0.72(0.47, 1.09)	0.80(0.49, 1.30)
12–23 months	60	40	0.39(0.28, 0.57)	**0.51(0.33, 0.77)** ^**a**^
24–59 months	126	201	1	1
Sex of the child				
Female	127	247	1.46(1.07, 1.99)	1.18(0.82, 1.70)
Male	103	292	1	1
Family size (mean)				
≤ 4	182	205	1	1
> 4	48	334	0.16(0.11, 0.23)	**0.13(0.09, 0.21)** ^**a**^
enrolled in the PSNP				
Yes	41	154	0.54(0.37, 0.79)	**0.63(0.40, 0.99)** ^**a**^
No	189	385	1	1
Father educational status				
Unable to read & write	140	287	0.56(0.26, 1.21)	**0.25(0.09, 0.66)**
Able to read and write	77	237	0.38(0.17, 0.82)	0.14(0.05, 0.37)
Above primary education	13	15	1	1
Husband occupation				
Farmer	7	29	1	1
Daily labourer	233	510	1.81(0.78, 4.20)	1.39(0.45, 4.26)
Maternal occupation				
Housewife	129	256	1.49(1.08, 2.06)	1.43(0.97, 2.10)
Daily labourer	13	22	1.75(0.85, 3.63)	2.33(0.90, 6.02)
Farmer	88	261	1	1
CDDS				
Poor (> 4 food groups)	39	52	1.91(1.22, 2.99)	**2.99 (1.67, 5.35)** ^**a**^
Good (≥4 food groups)	191	487	1	1
History of ANC follow-up				
Yes	201	496	1	1
No	29	43	1.66(1.01, 2.74)	1.29 (0.68, 2.48)
Birth interval				
< 2 years	62	221	0.53(.38, 0.75)	0.79(0.53, 1.19)
≥ 2 years	168	318	1	1
Vaccinated				
Yes	224	496	1	1
No	6	43	0.31(0.13, 0.74)	0.43(0.16, 1.18)

^a^Significant during multivariable analysis (*p* < 0.05), 1 = Reference, *AOR* adjusted odd ratio, *COR* crude odd ratio, *CDDS* child dietary diversity score

Children from divorced and separated women were 3.3 times (AOR: 3.33, 95% CI (1.71, 6.45)) more likely to develop wasting as compared to married. Children aged 24–59 months were 49% (AOR: 0.51, 95% CI (0.33, 0.77)) less likely to develop wasting as compared to 12–23 months of age. Also, children from smaller family sizes were 87% (AOR: 0.13, 95% CI (0.09, 0.21)) less likely to develop wasting.

Children who had a father above the primary educational level were 75% (AOR: 0.25, 95% CI (0.09, 0.66)) less likely to be wasted as compared to unable to read and write. Children from households enrolled with PNSP were 37% (AOR: 0.63, 95% CI (0.40, 0.99)) less likely to develop wasting as compared to families not covered with PSNP. In addition to this, children with low DDS were 3 times (AOR: 2.99, 95% CI (1.67, 5.35)) more likely to develop wasting.

## Discussion

In Ethiopia, still, undernutrition is a significant public health problem. Personal and community-level factors were significant determinants of childhood under-nutrition [8, 22]. In this study, the overall prevalence of wasting was 29.9% (95% CI: 26.6, 33.2%). Here, the prevalence of wasting is higher than the national prevalence reported by EDHS 2016 (10%) [8]. On the other hand, the finding is low as compared to the studies conducted at Wondogenet District, Sidama Zone, Southern Ethiopia (15.7%) [17] but comparable to the study conducted at Jigjiga Town, Somalia Regional State, Ethiopia [23]. The possible reasons might be the difference in the data collection period, socioeconomic status of parents, cultural difference, knowledge related to child feeding, and dietary habit.

Here, being in PSNP alone reduce the rate of wasting by 46%. In Ethiopia, enrolment into PSNP is based on household assets or wealth depletion; the program is designed for the poor to halt this problem. Those children from households with good assets or wealth were less likely to be wasted. These households might not have the risk of food self-sufficiency or acute shortage of foods. So, building household assets are very important by expanding this PSNP since socioeconomic status is one of the important prognosticators of an individual’s health status [24, 25]. Generally, the wealth status and possession score are used to assess household assets depletion based on the ownership status of selected household items [26, 27].

As a result, children from households enrolled with PSNP were 37% (AOR: 0.63, 95% CI (0.40, 0.99)) less likely to develop wasting as compared to families without PSNP which is similar to other studies conducted in Bangladesh and Cote d’Ivoire [27, 28]. This may be due to the strong relationship between low socioeconomic status and nutritional status. So, those households with PSNP were eligible due to the depleted household asset and/or poor agriculture productivity.

Children from divorced and separated women were 3.33 times (AOR: 3.33, 95% CI (1.71, 6.45)) more likely to develop wasting as compared to married women. This finding is in line with a study conducted in Dabat Town, North Gondar, Ethiopia [29]. On the other hand, this finding differed from the study result from Nakaseke and Nakasongola District, Uganda; most of the wasted children were from mothers who were married [30]. The possible justification might be household assets will deplete after divorce and being a female-headed household is a risk for economic burden.

Children who had a father above the primary educational level were 75% (AOR: 0.25, 95% CI (0.09, 0.66)) less likely to be wasted as compared to unable to read and write. The finding is similar to the study conducted in Jigjiga Town, Somali Regional State, Ethiopia [23]. The possible reason might be educational status associated with good feeding practice, better income, and asset building.

Children aged 24–59 months were 49% (AOR: 0.51, 95% CI (0.33, 0.77)) less likely to develop wasting as compared to 12–23 months of age. This is comparable with the national demographic and health survey of Ethiopia and a study conducted in Wondogenet District, Sidama Zone, Southern Ethiopia [8, 17]. Because, as the age increases the nutritional requirement increases, reduction of child care, discontinuation of breastfeeding, being pregnant of the mother, and poor dietary practice.

The other factor significantly associated with wasting was the family size. Children from smaller family sizes were 87% (AOR: 0.13, 95% CI (0.09, .21)) less likely to develop wasting like a study conducted in Jimma Zone, Southwest, Ethiopia [31] and Sebeta, Hawas District, Oromia, Ethiopia [32]. The possible reasons may be due to large family size with low socioeconomic status, which leads to inadequate intake, monotonous diet, a diet with poor nutrient density, inappropriate feeding practices, lack of hygienic practices, and illness. All these will lead to failure to take and absorb essential nutrients for growth & development [14] and may be due to household asset depletion.

In addition to this, children with low DDS were 3 times (AOR: 2.99, 95% CI (1.67, 5.35)) more likely to develop wasting. The dietary habit is the major factor, according to the systematic review and meta-analysis was done in Ethiopia [33]. The dietary habit, practice, and density highly, influenced by the socio-economic status of households which directly affect the nutritional status of the child.

The study was limited to link wasting to specific macronutrient deficiency, difficult to establish a possible causality, and recall-related problems (like a child date of birth and history of dietary intake). In addition to this, the study did not include clinical assessment and did not detect the subclinical condition.

## Conclusions

Generally, wasting was a severe public health problem. Being in the PSNP families had a significant contribution to the reduction of wasting or thinness among children independently. Generally, factors associated with wasting were marital status, being on the PSNP family, family size, the age of the child, and child DDS after adjusting/running all other variables.

Based on the finding, stakeholders should have to continue and expand the PSNP with the existed nutrition and other health-related strategies/programs to tackle wasting. Also, health professionals shall have to give health and nutrition education and counselling based on the age of the child, maternal educational status, and households PSNP enrolment status through other child and reproductive health care services.

## Data Availability

All the important data found in the manuscript.

## Abbreviations

AOR: Adjusted odds ratio
BMI: Body mass index
COR: Crude odds ratio
DDS: Dietary diversity score
HEWs: Health extension workers
PSNP: Productive safetynet program
SD: Standard deviation
SPSS: Statistical packages for social sciences
WHZ: Weight for height Z score
WAZ: Weight for age Z score
WHO: World Health Organization
